# Joint effect of maternal pre-pregnancy body mass index and folic acid supplements on gestational diabetes mellitus risk: a prospective cohort study

**DOI:** 10.1186/s12884-023-05510-y

**Published:** 2023-03-23

**Authors:** Minyu Li, Lijiang Wang, Zhanhui Du, Qianqian Shen, Lu Jiang, Lun Sui, Nan Zhang, Hong Wang, Guoju Li

**Affiliations:** 1 Public Health School, Medical College of Qingdao University, Qingdao, Shandong Province P.R. China; 2 Qingdao Women and Children’s Hospital, Qingdao University, Qingdao City, Shandong Province P.R. China; 3 School of Public Health, Peking university, Beijing, 100191 China; 4 Qingdao Women and Children’s Hospital, Qingdao University, No.6 Tongfu Road, Qingdao, 266000 China

**Keywords:** Pre-pregnancy body Mass Index, Folic acid supplements, Gestational diabetes Mellitus, Interaction

## Abstract

**Background:**

The joint effect of folic acid (FA) supplements and maternal pre-pregnancy body mass index (BMI) on gestational diabetes mellitus (GDM) has not been fully addressed. This study aimed to examine the joint effect of FA supplements and pre-pregnancy BMI on GDM.

**Methods:**

Pregnant women at 4 to 14 weeks of gestation (n = 3186) were recruited during their first prenatal visit in Qingdao from May 1, 2019, to June 27, 2021. The main outcome was GDM at 24–28 weeks’ gestation. Screening was based on 75 g 2-hour oral glucose tolerance (OGTT), a fasting glucose ≥ 5.1 mmol/L, or a 1-hour result ≥ 10.0 mmol/L, or a 2-hour result ≥ 8.5 mmol/L. The interactive effect of FA supplements and pre-pregnancy BMI on GDM was examined using logistic regression analysis and ratio of odds ratios (ROR) was used to compare subgroup differences.

**Results:**

Overall, 2,095 pregnant women were included in the analysis, and GDM incidence was 17.76%. Compared with women with pre-pregnancy BMI lower than 25.0 kg/m^2^ and FA-Sufficient supplements ≥ 400 µg/day (FA-S) population, the adjusted odds ratios (aORs) of FA-S and FA-Deficiency supplements < 400 µg/d (FA-D) were 3.57 (95% confidence interval [CI]: 2.02–6.34) and 10.82 (95% CI: 1.69–69.45) for the obese women (BMI ≥ 30.0 kg/m^2^), and the aORs of FA-S and FA-D were 2.17 (95% CI: 1.60–2.95) and 3.27 (95% CI: 1.55–6.92) for overweight women (25.0 kg/m^2^ ≤ BMI < 30.0 kg/m^2^). However, the risk of GDM did not differ significantly between the FA-D and the FA-S group in pre-pregnancy obese women (ROR = 2.70, 95%CI: 0.47–2.30), or overweight women (ROR = 0.66, 95%CI: 0.30–1.49). After further stratification of FA supplementation time, F-D and FA-S in obese women showed an interaction when FA supplement intake time < 3 months. However, there was no significant difference between subgroups (ROR = 1.63, 95% CI: 0.37–7.04).

**Conclusion:**

Maternal pre-pregnancy BMI was associated with the incidence of GDM, the dose of FA supplementation from pre-pregnancy to early pregnancy was not found to be related to the incidence of GDM. The dosage of FA supplement was not associated with GDM irrespective of maternal pre-pregnancy BMI.

## Introduction

Gestational diabetes mellitus (GDM) is one of the most common pregnancy complications worldwide [1–3]. Hyperglycemia during pregnancy is linked to adverse pregnancy outcomes, such as neonatal adiposity, macrosomia, large for gestational age, caesarean section and shoulder dystocia [4–6]. It also has long-term negative effects on women and their offspring [7–10]. Despite extensive public health efforts, the prevalence of GDM remains high around the world and continues to rise at an alarming rate, imposing an immense burden on global healthcare services [11–13]. This increase has been especially noticeable in China, and previous research showed the incidence of GDM had reached 17.42% in 2018–2019[14]. In view of the impact of this condition, there is an urgent need to identify modifiable risk factors.

Folic acid (FA) supplements are recommended before and during pregnancy around the world. Periconceptional consumption of FA, or multivitamins that contain FA, reduces the risk of neural tube defects (NTDs)[15, 16]. Since the beneficial effect of FA was well established, the link between daily FA supplements and GDM remains controversial. Recent reports suggest that the incidence of GDM may be increased in women who take FA supplements during pregnancy [17, 18]. However, a cohort study showed that a higher intake of habitual FA from supplements before pregnancy was significantly associated with a lower risk of GDM [19]. Experimental studies have shown that high-dose FA supplements throughout pregnancy may lower blood homocysteine levels and therefore protect against oxidative stress [20], which is known to contribute to endothelial dysfunction and insulin resistance [21]. Homocysteine concentrations have also been strongly linked to risk of GDM among pregnant women [22].

Previous studies have identified pre-pregnancy body mass index (BMI) as an important risk factor for GDM [23]. The effect of FA supplements or dietary folate intake on GDM may vary with maternal pre-pregnancy BMI. Studies have reported that there is an inverse interaction between pre-pregnancy BMI and serum folate levels. Obese individuals may be at risk of folate deficiency even after controlling for dietary and supplementary intake of FA [24–27]. Several potential mechanisms have been suggested for relative folate deficiency in obese women, such as chronic inflammation and hyperinsulinemia [28].

However, the joint effect of pre-pregnancy BMI and FA supplements on GDM is unclear. To address this gap in knowledge, this cohort study aimed to examine the interaction between FA supplements and pre-pregnancy BMI on the risk of GDM. It also, considered the time of the supplementation, to provide favorable evidence for effective antenatal nutritional interventions.

## Methods

### Data sources and cohort

This was a prospective cohort study of pregnant women at 4 to 14 weeks of gestation from May 1, 2019, to June 30, 2021. We recruited a total of 3,186 pregnant women. Details of the study have been described previously [29]. All the pregnant women in the study were from the Qingdao Women and Children’s Hospital Health Cohort, which is a prospective cohort designed to determine the impact of maternal dietary, environmental and lifestyle exposures on the health of pregnant women and their offspring. At registration, questionnaire-based interviews were used to gather information on social demographic status, reproductive variables, family history of diseases, the use of supplementation, lifestyle factors, and illnesses. Women responded to a semiquantitative food frequency questionnaire (FFQ) [30], where they reported the frequencies at which they had consumed a specific portion of each of the 25 food or food group items during the past six months. They also reported their use of dietary supplements, including the brand, dose, and frequency of use. The daily food consumption and nutrient intakes in the FFQ were calculated according to the China Food Composition Tables (6th edition) [31]. Throughout the follow-up visits during mid-pregnancy and late pregnancy, information on lifestyle, dietary intake, and the use of supplements was acquired. Participation in the study was entirely voluntary, and each study participant provided written informed consent.

### Assessment of FA supplements and pre-pregnancy BMI

Participants were asked for information about FA supplements at enrollment. This included brand, daily doses, and the time of supplements. In this study, taking sufficient FA supplements (FA-S) was defined as taking either an FA specific supplement or FA-containing supplements of at least 400 µg/day, deficiency of FA supplements was defined as taking either FA specific supplement or FA-containing supplements less than 400 µg/d (FA-D) [32]. Pregnant women were divided into underweight/normal (pre-pregnancy BMI < 25.0 kg/m^2^), overweight (25.0 kg/m^2^ ≤ pre-pregnancy BMI < 30.0 kg/m^2^) and obese (pre-pregnancy BMI ≥ 30.0 kg/m^2^) [33].

### Diagnosis of GDM

In line with the Ministry of Health of China’s Diagnostic Criteria for Gestational Diabetes Mellitus (WS311-2011), all participants were screened for GDM using a 75 g oral glucose tolerance test (OGTT) at 24 to 28 weeks’ gestation [34]. The criterion cut-off values were consistent with the International Association of Diabetes and Pregnancy Study Groups Consensus Panel recommendations [35]. A diagnosis of GDM can be made if any of the following values in the 75 g OGTT were met or exceeded: 0-hour (fasting plasma glucose) ≥ 5.1mmol/L, 1-hour ≥ 10.0 mmol/L, or 2-hour ≥ 8.5 mmol/L.

### Assessment of covariates

The questionnaire information included, social demographic characteristics, living environment, personal and family history of diseases, dietary content, and anthropometric information. When participants were enrolled, we measured weight, height, waist circumference, hip circumference, and blood pressure. The pre-pregnancy BMI was calculated by dividing self-reported weight before pregnancy in kilograms by the square of height in meters measured at enrollment. Smoking activities were divided into active and passive smoking (second-hand smoke exposure). Passive smoking was divided into pre-pregnancy contact, pregnancy contact, and pre-pregnancy to pregnancy exposure. Drinking was defined those consuming alcohol more than three times per week. We also identified whether participants took vitamin B_12_ supplements.

### Statistical analysis

Numerical variables were expressed as mean ± standard deviation (SD). Frequency of category variable expressed in percentage [n (%)]. Maternal characteristics were compared by FA supplements use status using ANOVA for continuous variables and Chi-square test for categorical data. Logistic regression analysis was performed and odds ratios (OR, with 95% confidence intervals [CI]) were calculated to evaluate the risk associated with GDM. We used a binomial logistic regression model to estimate odds ratios (ORs) and 95% CIs of incidence of GDM in by category of folate intake (sufficient and deficiency [≥ 400 ug/day and < 400 ug/day] for total and supplemental folate intake, and across quartiles of food folate intake). Linear trends of GDM risk across categories of folate intake were examined by fitting the models using the median intake of each category of folate intake as a continuous variable.

We examined interaction effects on the multiplicative scale. For multiplicative interaction, we calculated two-sided *P-* values to assess the significance of each product term in the logistic regression models and compared the ORs for pre-pregnancy BMI across FA supplement doses. To clarify the relationship further, we carried out a stratified analysis by the intake time for FA supplements to determine the joint effect of pre-pregnancy BMI and FA supplements level on GDM in different groups. We hypothesized that the effect estimates would be greater for the association of obesity with FA-D than FA-S, and tested this by computing the ratio of odds ratios (ROR). A *P*-value of 0.05 or less was considered significant, and an ROR > 1.00 and the 95% CI does not contain 1.00 signified a statistically significant difference between two ORs [36]. All the data were analyzed using SAS 9.4 software.

## Results

**Fig. 1:**
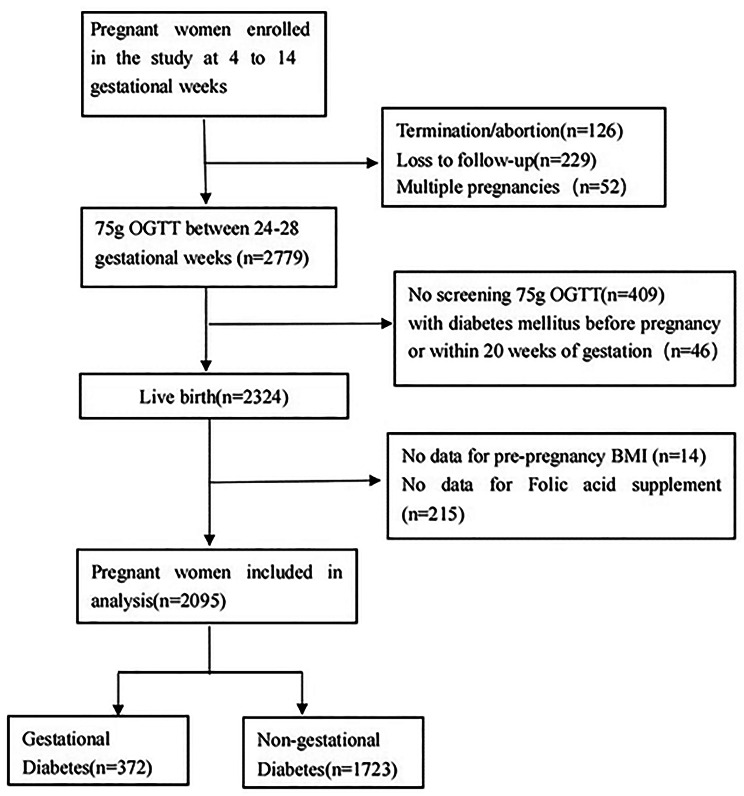
Flow chart of the screening process for the selection of eligible participants

In the cohort of Qingdao Women and Children’s Hospital, we recruited 3,186 pregnant women, 1,091 were excluded according to the exclusion criteria, and finally 2,095 pregnant women were included in the data analysis. The exclusion criteria were: (1) multiple pregnancy (n = 52); (2) termination or abortion (n = 126), loss to follow-up before 24–28 gestational weeks (n = 229), or no 75 g oral glucose tolerance test (OGTT) information (n = 409); (3) incomplete or missing information on height and weight before pregnancy (n = 14); (4) incomplete or missing information about FA supplements, with unclear doses and unclear duration(n = 215); and (5) History of diabetes (n = 26) and with diabetes mellitus before pregnancy or within 20 weeks of gestation(n = 30) (Fig. 1). The incidence of GDM among the 2,095 women with singleton births was 17.76% (n = 372). Overall, 186 (8.88%) of the participants had either not taken any FA supplements or their daily supplement consumption was less than 400 µg before pregnancy and in the first trimester. The proportion of women consuming less than 400 µg/day of FA supplements was higher among those with pre-pregnancy BMI ≥ 30.0 kg/m², but the difference was not significant (*P* > 0.05). Intake of vitamin B_12_ supplements was higher among pregnant women with FA ≥ 400 µg/day (*P* < 0.05) (Table 1).

**Table 1:** Demographic characteristics of the FA supplement use status (n = 2095)

	FA-D(n = 186,8.88%)	FA-S (n = 1909,91.12%)	*p-value*
Age (years)			0.374
< 35	160(86.02)	1626(85.18)	
≥ 35	26(13.98)	283(14.82)	
Pre-pregnancy BMI (kg/m^2^)			0.405
< 25.0	147(79.03)	1573(82.40)	
25.0 ≤ BMI < 30.0	34(18.28)	279(14.61)	
≥ 30.0	5(2.69)	57(2.99)	
Education(years)			0.748
Under the high school	26(13.98)	288(15.09)	
High school and above	160(86.02)	1621(84.91)	
Monthly income (¥)			0.320
< 5000	46(24.73)	554(29.02)	
≥ 5000	140(75.27)	1355(70.98)	
Parity			0.060
0	123(66.13)	1387(72.66)	
≥ 1	63(33.87)	522(27.34)	
Smoking			
Active smoking			0.606
No	175(94.09)	1803(94.45)	
Yes	11(5.91)	106(5.55)	
Passive smoking			0.850
No	159(85.48)	1648(86.33)	
pre-pregnancy	12(6.45)	108(5.66)	
after pregnancy	0(0.00)	7(0.37)	
pre-pregnancy to pregnancy	15(8.06)	146(7.65)	
Drinking			0.653
No	182(97.85)	1849(96.86)	
Yes	4(2.15)	60(3.14)	
Family history of diabetes mellitus			0.310
No	132(70.97)	1433(75.07)	
Yes	53(28.49)	454(23.78)	
Unclear	1(0.54)	22(1.15)	
Family history of hypertension			0.531
No	106(56.99)	1007(52.75)	
Yes	75(40.32)	853(44.68)	
Unclear	5(2.69)	49(2.57)	
Fertilization way			0.618
Natural conception	169(90.86)	1711(89.63)	
Non-natural conception	17(9.14)	198(10.37)	
History of GDM			0.677
No	181(97.31)	1841(96.44)	
Yes	5(2.69)	68(3.56)	
Gestational weight gain (kg)	1.18 ± 2.70	1.21 ± 2.78	0.811
Vitamin B_12_ supplements			**< 0.001**
No	163(87.63)	887(46.46)	
Yes	23(12.37)	1022(53.54)	

Data are presented as n (%), mean ± SD, *P*-values are determined by chi-square, independent t-test

This study evaluated folate intake from supplements and food, both together (i.e., total folate) and separately, as the exposures of interest (Table 2). After adjustment for age, pre-pregnancy BMI, education level, monthly income, passive smoking, drinking, family history of diabetes mellitus, mode of fertilization, history of GDM, and the use of vitamin B_12_ supplements, the ORs of GDM across increasing quartiles of food FA intake were 1.00 (reference), 1.02 (95% CI: 0.72–1.44), 1.08 (0.77–1.52), and 0.86 (0.60–1.23), respectively (*P*_trend_ = 0.980). The ORs of GDM across increasing quartiles of total FA intake were 1.00 (reference), 0.78 (95% CI: 0.56–1.10), 0.82 (0.58–1.17), and 0.84 (0.57–1.24), respectively (*P*_trend_ = 0.919). Sufficient total folate intake (≥ 400 ug/day) was a OR of GDM of 1.25 (95% CI: 0.64–2.43) (*P* = 0.518) compared with a deficient intake (< 400 ug/day). Food folate intake was not associated with GDM risk.

**Table 2:** Odds ratio (95% confidence interval) of GDM according to folate intake

	GDM/pregnancy	Crude OR	Adjusted OR
Food folate(ug/day)			
Q1(28–130)	116/524	1.00(ref.)	1.00(ref.)
Q2(131–206)	84/525	1.03(0.74–1.45)	1.02(0.72–1.44)
Q3(207–308)	88/523	1.16(0.83–1.62)	1.08(0.77–1.52)
Q4(308–1572)	84/523	0.94(0.66–1.33)	0.86(0.60–1.23)
*P*_trend_		0.846	0.980
Supplemental folate(ug/day)			
< 400	35/186	1.00(ref.)	1.00(ref.)
≥ 400	337/1909	0.93(0.63–1.36)	0.86(0.58–1.30)
*P*		0.692	0.483
Total folate(ug/day)			
Q1(38–602)	102/524	1.00(ref.)	1.00(ref.)
Q2(603–833)	80/527	0.80(0.58–1.12)	0.78(0.56–1.10)
Q3(834–1001)	95/522	0.92(0.68–1.26)	0.82(0.58–1.17)
Q4(1001–2296)	95/522	1.01(0.73–1.39)	0.84(0.57–1.24)
*P*_trend_		0.762	0.919
Total folate(ug/day)			
< 400	13/78	1.00(ref.)	1.00(ref.)
≥ 400	359/2017	0.84(0.44–1.60)	1.25(0.64–2.43)
*P*		0.595	0.518

Q, quartile. Adjusted OR: adjusted for age, pre-pregnancy BMI, education level, monthly income, passive smoking, drinking, family history of diabetes mellitus, fertilization way, history of GDM, the use of vitamin B_12_ supplement

Table 3 shows the effects of pre-pregnancy BMI and daily FA supplement on GDM. Compared with pre-pregnancy BMI < 25.0 kg/m^2^, pregnant women who were overweight (OR = 2.38, 95% CI: 1.81–3.14)) and obese (OR = 3.61, 95% CI: 2.13–6.12) had increased risk of GDM (*P* < 0.05). Table 3 also shows the aORs, and similar results were observed in the association between pre-pregnancy BMI and GDM in the adjusted model (*P* < 0.05). Compared with FA intake < 400 µg /day, FA intake ≥ 400 µg /day had no a significant association with GDM, regardless of adjustment (*P* > 0.05).

**Table 3:** Odds ratio (95% confidence interval) of GDM according to pre-pregnancy BMI and FA supplement use doses as categorical

	N(%)	Crude OR	*P-value*	Adjusted OR	*P-value*
BMI (kg/m^2^)					
< 25.0	1720(82.10)	Ref	-	Ref	-
25.0≤ BMI < 30.0	313(14.94)	2.38(1.81–3.14)	**< 0.001**	2.28(1.71–3.03)	**< 0.001**
≥ 30.0	62(2.96)	3.61(2.13–6.12)	**< 0.001**	3.91(2.27–6.74)	**< 0.001**
FA intake					
< 400 µg /day	186 (8.89)	1.12(0.77–1.65)	0.550	1.49(0.53–4.23)	0.453
≥ 400 µg /day	1909(91.11)	Ref	-	Ref	-

Adjusted OR: adjusted for age, education level, monthly income, passive smoking, drinking, family history of diabetes mellitus, fertilization way, history of GDM, the use of Vitamin B_12_ supplement. Variables with statistical significance were shown in boldface

To further determine the joint effect of FA supplements and pre-pregnancy BMI on GDM risk (Table 4), we divided pregnant women into six groups by both pre-pregnancy BMI and FA supplement levels [Group 1: FA-S and BMI < 25.0 kg/m^2^; Group 2: FA-D and BMI < 25.0 kg/m^2^; Group 3: FA-S and BMI (25.0 kg/m^2^ -30.0 kg/m^2^); Group 4: FA-D and BMI (25.0 kg/m^2^ -30.0 kg/m^2^); Group 5: FA-S and BMI ≥ 30.0 kg/m^2^; Group 6: FA-D and BMI ≥ 30.0 kg/m^2^]. Compared with Group 1, the aOR of Group3, 4, 5 and 6 were 2.17 (95% CI: 1.60–2.95), 3.27 (95% CI: 1.55–6.92), 3.57 (95% CI: 2.02–6.34) and 10.82 (95% CI: 1.69–69.45) (all *P* < 0.05). The ROR value was 2.70 (95% CI: 0.47–2.30) for the two subgroups with different FA doses in the population with BMI ≥ 30.0 kg/m^2^ and 0.66 (95%CI: 0.30–1.49) for those with BMI of 25.0 kg/m^2^ -30.0 kg/m^2^. The RORs and the corresponding lower boundaries of the confidence intervals were both greater than 1, and there is therefore no good evidence to support a different risk effect with different levels of FA supplementation.

**Table 4:** Interaction analysis of pre-pregnancy BMI and FA supplement intake dose on the risk of GDM

Interaction	Crude OR	*P*-value	Adjusted OR	*P*-value
FA-S*BMI (< 25.0 kg/m^2^)	Rf		Rf	
FA-D*BMI (< 25.0 kg/m^2^)	0.95(0.59–1.54)	0.831	0.99(0.60–1.66)	0.997
FA-S*BMI (25.0 kg/m^2^≤ BMI < 30.0 kg/m^2^)	**2.29(1.71–3.07)**	**< 0.001**	**2.17(1.60–2.95)**	**< 0.001**
FA-D*BMI (25.0 kg/m^2^ ≤ BMI < 30.0 kg/m^2^)	**3.11(1.52–6.36)**	**0.002**	**3.27(1.55–6.92)**	**0.002**
FA-S*BMI (≥ 30.0 kg/m^2^)	**3.32(1.91–5.79)**	**< 0.001**	**3.57(2.02–6.34)**	**< 0.001**
FA-D*BMI (≥ 30.0 kg/m^2^)	**8.54(1.42–51.38)**	**0.019**	**10.82(1.69–69.45)**	**0.012**

Adjusted OR: adjusted for age, education level, monthly income, passive smoking, drinking, family history of diabetes mellitus, fertilization way, history of GDM, the use of vitamin B_12_ supplement. Variables with statistical significance were shown in boldface

**Fig. 2:**
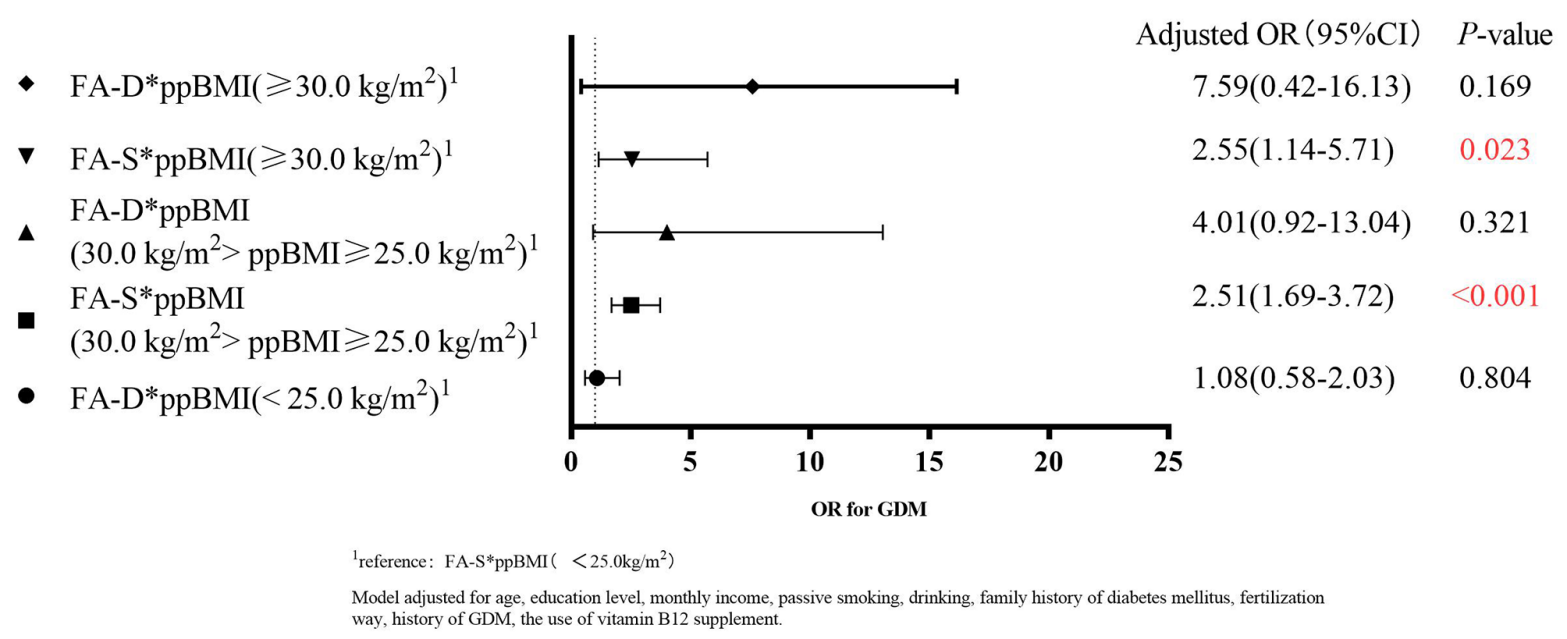
Interaction analysis of pre-pregnancy BMI and FA supplement intake dose on the risk of GDM (FA supplement intake time ≥ 3 months)

**Fig. 3:**
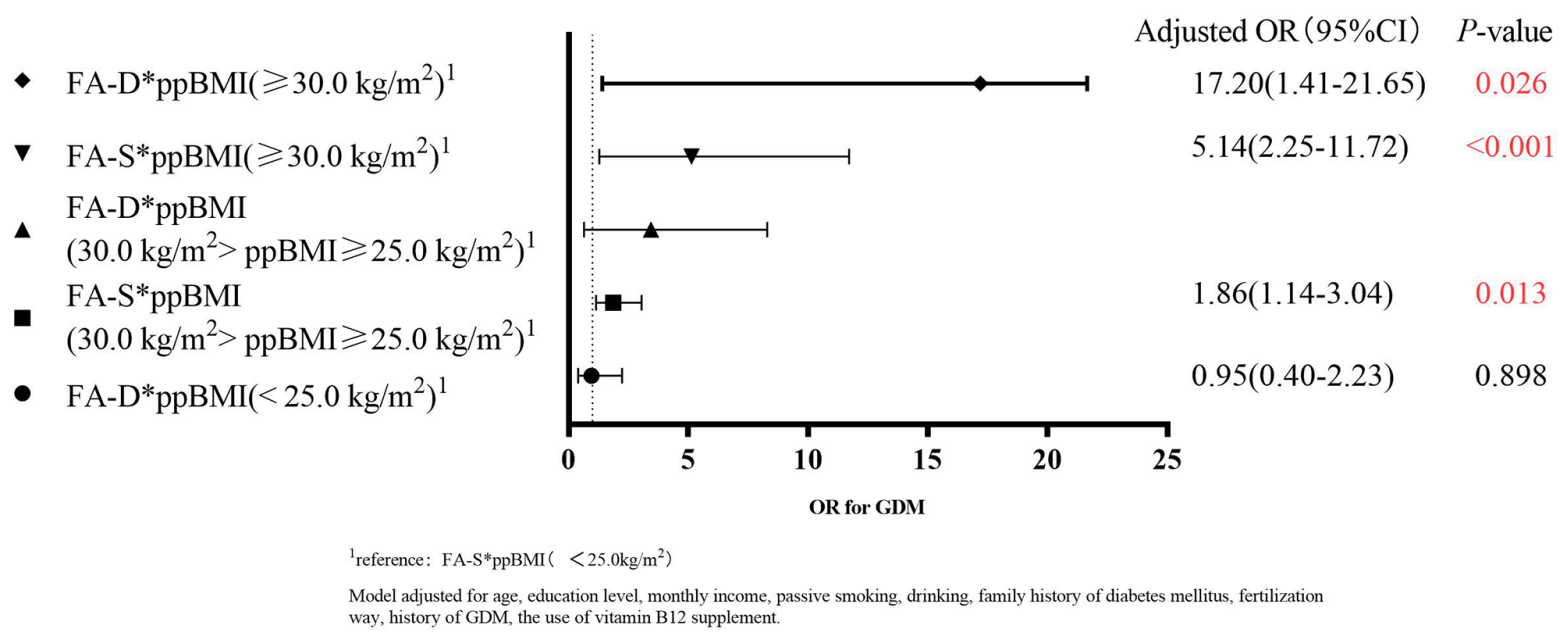
Interaction analysis of pre-pregnancy BMI and FA supplement intake dose on the risk of GDM (FA supplement intake time < 3 months)

To clarify the effect of FA supplements and pre-pregnancy BMI on GDM, we carried out stratified analyses by the time of FA intake (Figs. 2 and 3). We calculated the ROR = 3.35, 95% CI: 0.68–16.49 between the FA-D (OR = 17.20, 95% CI: 1.41–21.65) and FA-S (OR = 5.14, 95% CI: 2.25–11.72) subgroups in obese (BMI > 30.0 kg/m^2^) women taking FA supplements < 3 months. There was no statistical difference between FA and the risk of GDM after stratification by FA intake time.

## Discussion

Maternal pre-pregnancy BMI was associated with the incidence of GDM, but the dose of FA supplementation from pre-pregnancy to early pregnancy was not related to the incidence of GDM, irrespective of maternal pre-pregnancy BMI. After subgrouping, the interaction was not statistically significant, but this may have been due to the limited number of FA-D individuals (186/2,095, 8.88%) in our cohort. This is probably mostly due to recent work by the Chinese government [37], including the provision of public health services and the distribution of free FA supplements, which have boosted the percentage of pregnant women who use FA supplements throughout pregnancy.

FA is an important pregnancy nutrient for its protective effects against birth defects.

The Chinese Government places a high priority on the prevention of congenital anomalies by promoting FA supplementation. Plenty of countries have proposed that flour be fortified with folate for the prevention of NTDs [38, 39]. However, China currently has no policies on mandatory folate fortification [37], and the prevention of birth defects in China is mainly achieved through the promotion of FA supplements for women of the right age, and the distribution of free folic acid supplements [40, 41].

An article published in the journal *Diabetes Care* shows that average total folate intake (i.e., ≥ 400 ug/day) was significantly associated with lower risk of GDM This association was entirely driven by folate from supplements, and food folate was not associated with GDM risk [19]. This consistent with our finding that folate intake from food was much lower than from supplements and thus may be insufficient to achieve an effect against GDM. Folate, in supplements is also more bioavailable than food folate [42]. Other studies have also reported that supplemental folate has stronger associations with relevant health outcomes than food folate [43, 44]. We therefore only considered the dosage of FA supplements in further analyses.

Previous studies evaluating the association of FA supplementation before or during pregnancy with GDM risk have conflicting results [18]. A large prospective cohort (n = 20,199) showed that habitual intake of FA supplements preconception was inversely associated with GDM risk in the United States [19]. However, a prospective Chinese study of 326 pregnant women showed that high-dose FA supplementation in early pregnancy was associated with an increased risk of GDM [45]. This discrepancy might be due to a smaller sample size in the latter study. Consistent with this, a prospective cohort study showed that daily FA supplementation in the first trimester was positively associated with GDM risk [17]. However, it is difficult to interpret this finding because details of the research methods and results were not reported. Cueto and colleagues [46] found no clear association between preconception FA use and diabetes diagnosis, and our result is consistent with this. The relationship between maternal FA intake and GDM therefore needs further examination through larger cohort studies.

Obesity affects short-term folate pharmacokinetics through diminished uptake of orally administered FA. The low serum folate status associated with obesity may be due to a volumetric dilution of the blood in obese individuals and/or low folate intake in the obese population [47]. Another explanation may be that adiposity influences folate uptake by the intestinal epithelium [48, 28]. This suggests that FA may not be distributed freely in adipose tissue. An alternative explanation is that the reduction of the ratio of surface area to volume of mast adipocytes may limit the penetration rate [49].

Obesity also affects the metabolism of serum folate. A retrospective case-control study found that higher BMI in the first trimester was negatively correlated with serum folate levels in the third trimester [50]. Another possible explanation is that obesity can increase estrogen, which has been reported to be associated with decreased serum folate availability [51]. It is therefore plausible that pathways related to metabolic regulation may underpin the associations between BMI and serum folate.

Previous studies have suggested that different BMI levels may influence the effect of FA supplementation on disease. A case–control study found that the association between FA supplements and the NTDs risk was weaker in overweight/obese mothers than in underweight/normal weight mothers. This suggested that maternal BMI could affect the association between FA supplementation and the NTDs risk in offspring [52]. A retrospective cohort study [53] reported that the protective effect of FA supplements against preterm delivery (PTD) was reduced in women whose BMI was equal to or greater than 24.0 kg/m^2^. However, few articles have examined the relationship between FA and BMI on GDM. This study was therefore important in analyzing the interaction between FA and BMI and the relationship between FA, BMI and GDM.

One of the most interesting observations of this study is that the risk of GDM was increased in obese women regardless of adequate folate intake. However, the subgroup analysis found that there was no heterogeneity between the two groups, which meant that different intake doses of FA supplements would not affect the incidence of GDM. Prospective cohort studies in China have assessed the impact of FA supplement use on GDM with consideration of both doses and durations. One showed a U-shape relationship between duration of FA supplements and risk of GDM [45], and another suggested that long-term use of high-dose FA increased GDM risk [19]. We therefore also compared the interaction between the FA supplements and pre-pregnancy BMI, dividing the groups into FA taken for at least 3 months and less than 3 months. Risk of GDM in obese women with both deficient and sufficient FA intake was still higher than women with BMI < 25.0 kg/m^2^ and FA-S. There was no statistical association between FA supplement and the risk of GDM after stratification by FA intake time.

The biological mechanisms that underlie the modified association are complicated and remain unclear. However, our hypothesis could be partly supported by the theory that FA could inhibit homocysteine production [54, 55]. A previous study found that homocysteine concentrations declined as FA concentrations increased, as did the prevalence of hyperhomocysteinemia [56]. High concentrations of homocysteine are associated with insulin resistance [57, 58]. These findings suggest that FA might have a protective effect on GDM by reducing homocysteine concentration and improving insulin resistance. However, a higher BMI might decrease the levels of serum folate or dietary folate intake [59–61]. The combine effect of high pre-pregnancy BMI and low dose FA intake leads to greater homocysteine concentrations and reduced insulin resistance, resulting in GDM. Therefore, we suggest that plans for FA supplementation should vary with women’s BMI category.

Epigenetics is defined as alterations in the gene expression profile of a cell that are not caused by changes in the deoxyribonucleic acid (DNA) sequence [62]. Folate may affect the incidence of GDM by influencing epigenetics. Epigenetics is critical to normal genome regulation and development. One-carbon metabolism is required for epigenetic modifications because it provides methyl groups for the methylation of DNA, associated proteins, which requires an adequate supply of folate [63]. Periconceptional FA supplementation has been linked to epigenetic changes [64]. These epigenetic modifications, particularly DNA methylation, have been proposed as plausible mechanisms underlying associations between folate and various disease outcomes, including NTDs, cardiovascular disease, and cancer [65, 66].However, so far, there is no direct evidence that high dietary folate or folate intake will lead to abnormal DNA methylation, or to diabetes in pregnancy. This is because DNA methylation is part of a complex, highly regulated system. Further research is needed to clarify the relationship between folic acid, DNA methylation and GDM.

Our study has several advantages. Firstly, it was a prospective cohort study, which reduces the effects of selection or recall bias. We excluded women with hypertension or established diabetes to avoid information bias. Secondly, previous studies also shown that vitamin B_12_ in multivitamin supplements has an impact on the risk of GDM [67, 68]. We collected sufficient data to include various confounders in the adjusted analyses and matched for vitamin B_12_ as a confounder. This allowed us to assess the effects of the interaction of FA supplements alone with pre-pregnancy BMI on GDM. However, the study also had several limitations. First, FA exposure was determined by self-reported FA supplement use rather than plasma folate levels. Misclassification is therefore a possibility. However, significant efforts were made to ensure that reliable FA supplement use data were collected on time by trained medical personnel with meticulous follow-up. Self-reported FA intake from supplements has also previously been found to be correlated with plasma folate and is therefore regarded as a reliable indicator of folate exposure [56]. Second, we mainly analyzed daily intake of FA by pregnant women from pre-pregnancy to first trimester. The FA intake during the whole pregnancy was not analyzed, but our study is consistent with the recommended folic acid intake time in the Nationwide Folic Acid Supplementation Program of China [3]. Third, the relatively small sample size in our study also limited our ability to investigate the relationship between FA supplements and pre-pregnancy BMI at different levels.

In future, our research group will consider collecting biochemical data and analyzing the effects of serum folate and erythrocyte folate on GDM to further enrich the literature on the relationship between FA and GDM. Our findings should provide new perspectives to support the development of prevention strategies, and further studies should consider larger sample sizes, total time from pre-conception to post-conception, and sophisticated statistical methods to examine the relationship between FA supplements, pre-pregnancy BMI, and pregnancy disorders.

## Conclusion

Maternal pre-pregnancy BMI was associated with incidence of GDM. However, the dose of FA supplements from pre-pregnancy to early pregnancy was not related to GDM, irrespective of maternal pre-pregnancy BMI.

## Data Availability

Data are available from the corresponding author on reasonable request.

## Abbreviations

FA: folic acid
GDM: gestational diabetes mellitus
BMI: body mass index
NTDs: neural tube defects
PTD: preterm delivery
OGTT: oral glucose tolerance test CI:confidence intervals
OR: odds Ratio
aOR: adjusted odds Ratio
ROR: ratio of odds ratios
DNA: deoxyribonucleic acid.
